# Supplementary material for: Antibacterial property of lead telluride quantum dot layer fabricated on glass substrate
Source: PLoS One. 2025 Oct 16;20(10):e0334629. doi: 10.1371/journal.pone.0334629 (PMC12530537; doi:10.1371/journal.pone.0334629)
Supplement: S1 Fig — (PDF) [file pone.0334629.s001.pdf]

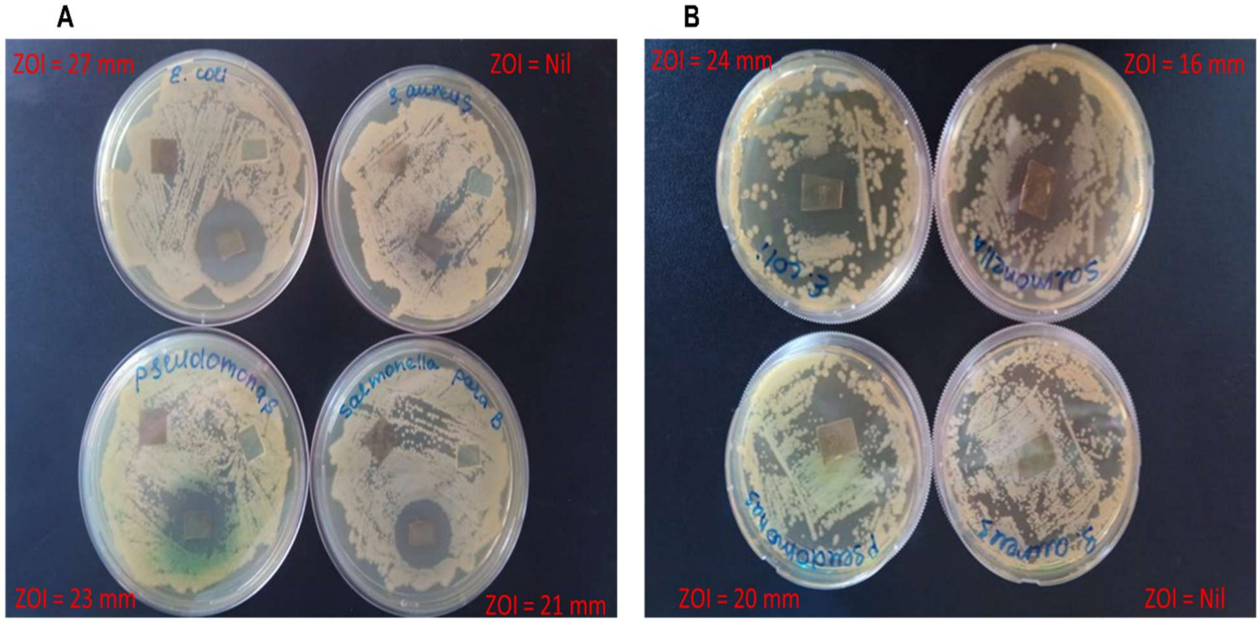

S1 Fig. **6.1 nm PbTe quantum dot layered substrates showing repeat of antibacterial effect** (A) First contact, (B) post autoclave.
