# Supplementary material for: Antibacterial property of lead telluride quantum dot layer fabricated on glass substrate
Source: PLoS One. 2025 Oct 16;20(10):e0334629. doi: 10.1371/journal.pone.0334629 (PMC12530537; doi:10.1371/journal.pone.0334629)
Supplement: S2 Fig — (PDF) [file pone.0334629.s002.pdf]

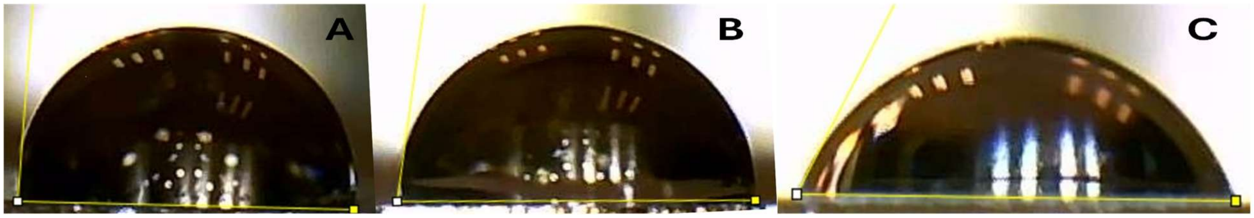

S2 Fig. **An example of the contact angle measurement on** (A) PbTe quantum dot layer with LE, (B) PbTe quantum dot layer without LE, and (C) blank control.
