# Supplementary material for: Antibacterial property of lead telluride quantum dot layer fabricated on glass substrate
Source: PLoS One. 2025 Oct 16;20(10):e0334629. doi: 10.1371/journal.pone.0334629 (PMC12530537; doi:10.1371/journal.pone.0334629)
Supplement: S3 Fig — (PDF) [file pone.0334629.s003.pdf]

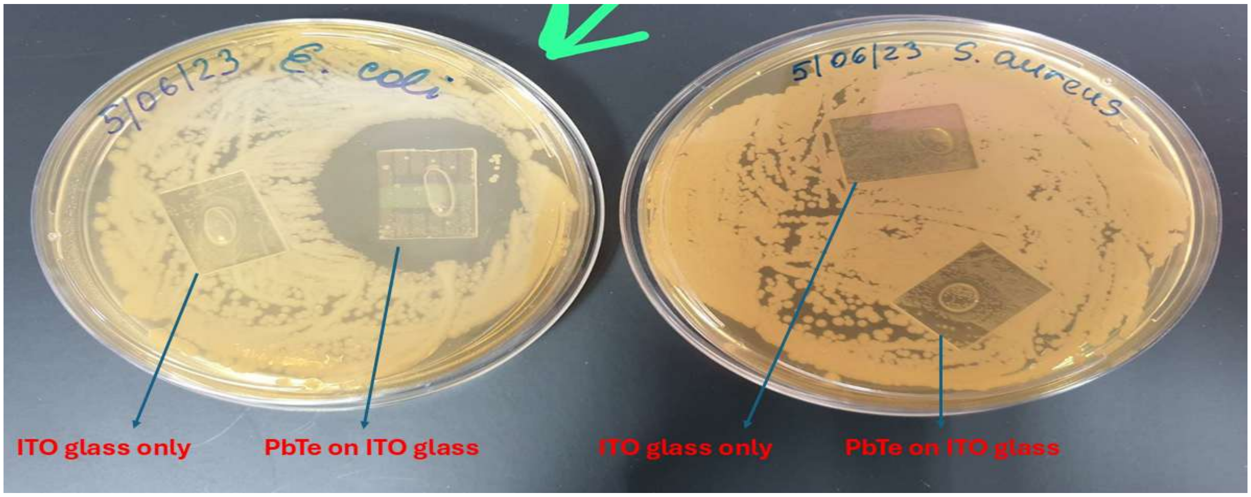

S3 Fig. **An experiment showing the effect of ITO glass on representatives of Gram negative and Gram positive bacteria** (A) *E. coli*, (B) *S. aureus.*
