# Supplementary material for: Antibacterial property of lead telluride quantum dot layer fabricated on glass substrate
Source: PLoS One. 2025 Oct 16;20(10):e0334629. doi: 10.1371/journal.pone.0334629 (PMC12530537; doi:10.1371/journal.pone.0334629)
Supplement: Attachment — Submitted filename: Comments - PONE-D-24-42235.docx [file pone.0334629.s004.docx]

The manuscript entitled "Antibacterial Property of Lead Telluride Quantum Dot Layer Fabricated on Glass Substrate " by Refael Minnes and co-workers reported the synthesis of PbTe QDs were synthesized using simple spin coating method method and their application as Antibacterial Property. However, this manuscript seems to be an unfinished work. In my opinion, more work should be done before resubmitting.

Authors can improve the article according to the following suggestions to make it suitable for publication in this journal.

1. Antibacterial properties of this material may not have been investigated due to its toxicity. On what basis, PbTe has been chosen for the study? The justification should be mentioned in the introduction section.

2. Why PbTe QD’s is above Titanium dioxide (TiO2), because it has proven to have antibacterial properties.

3. Introduction – Elaborate on the limitations of currently employed methods for antibacterial properties, supported by relevant references.

4. The novelty of this work should be highlighted.

4. I request the authors to look at all the figures and carefully edit them. They are not clear/not visible.

5. Consider providing a schematic diagram illustrating the mechanism of nanoparticle formation.

6. How about the effect of PbTe QD size on the antibacterial activity?

6. Discussion of the antibacterial activity must be cited with relevant literature. No previous study statistics were mentioned.

7. Delete some references that are not relevant to the article and mention the correct ones (example Reference [26, 35..]).
