# Supplementary material for: Antibacterial property of lead telluride quantum dot layer fabricated on glass substrate
Source: PLoS One. 2025 Oct 16;20(10):e0334629. doi: 10.1371/journal.pone.0334629 (PMC12530537; doi:10.1371/journal.pone.0334629)
Supplement: Attachment — Submitted filename: comment.docx [file pone.0334629.s006.docx]

**Manuscript Id: PONE-D-24-42235R1**

**Title: “Antibacterial Property of Lead Telluride Quantum Dot Layer Fabricated on Glass Substrate”**

**Respected Editor,**

Thank you for the opportunity to review the PLOS ONE manuscript The authors have revised the manuscript according to the comments and I recommend that the manuscript be accepted for publication.
