# Supplementary material for: Antibacterial property of lead telluride quantum dot layer fabricated on glass substrate
Source: PLoS One. 2025 Oct 16;20(10):e0334629. doi: 10.1371/journal.pone.0334629 (PMC12530537; doi:10.1371/journal.pone.0334629)
Supplement: Attachment — Submitted filename: Response_To_Reviewers_auresp_2.docx [file pone.0334629.s007.docx]

**Response to Reviewers’ Comments on Manuscript PONE-D-24-42235R1**

The Academic Editor

*PLOS ONE*

Dear Dr. Suresh,

Thank you for giving us the opportunity to submit a revision of the manuscript ‘Antibacterial Property of Lead Telluride Quantum Dot Layer Fabricated on Glass Substrate’ (PONE-D-24-42235R1) for publication in *Plos One.* The revision comments were each considered and the suggestions by the reviewers have also been implemented, with changes highlighted in the manuscript. Additionally, references 33, 34, and 47 have been added to consolidate data already provided in the initial draft. Please see below a point-by-point response to the reviewers’ comments. All page numbers refer to the revised manuscript file with tracked changes.

Review Comments for the Authors

**Reviewer 1**

**Comment 1:** R1#2: The authors did not understand the comment. Tempered glass and ITO may be similar in terms of transparency but not cost. The authors either know the results already or are reluctant to experiment with whether their ligand-based QD is sustainable.

**Author Response:** Thank you for the clarification. We have noted this in the discussion session in lines 246 to 249, proposing application-specific cost-benefit analysis for any scenarios where our layers may be considered for use.

**Comment 2:** R1#4: Spin coating for solar panels is not economically viable but an experimental proof of concept.

**Authors Response:** We thank you for this observation. The manuscript has been edited to reflect this in lines 246 to 249.

**Comment 3:** R1#5: Show the surface roughness values with and without TiO_2_.

**Authors Response:** Lyssenko et al., 2025 ([doi.org/10.1371/journal.pone.0317677](https://doi.org/10.1371/journal.pone.0317677.g001)) showed that controlling the surface roughness is important for achieving thin film monolayer of PbTe on ITO, reporting around a 61% reduction in surface roughness on ITO after layering with TiO_2_. This article citation has been added to our manuscript to justify the addition of TiO_2_ layer.

**Comment 4:** R1#8: If it is ionic diffusion as proposed, how do the authors prove the sustainability of the coating/coated material for any application?

**Authors Response:** Thank you for your comment. We note that lead chalcogenides have been suggested to show multiple modes of antibacterial action, including ionic diffusion. While our fabricated layers showed leaching with use, more permanent immobilization of the active material on substrates can be achieved for practical purposes, in which case alternative methods like ROS generation, which has also been proposed for Lead chalcogenides, would mediate the antibacterial activity.

**Comment 5:** Fig 2: R1 Version: If the SEM image corresponds to QDs on TiO_2_, where is the TiO_2_ layer/particles?

**Authors Response:** A detailed report on fabrication and characterization of the PbTe layers as used in our experiments was given in Lyssenko et al., 2025 ([doi.org/10.1371/journal.pone.0317677](https://doi.org/10.1371/journal.pone.0317677.g001)), including cross-section high resolution TEM image showing the layers of PbTe QDs, TiO_2_ and ITO.

**Comment 6:** Nowhere in the original version, the authors have claimed to observe a cuboid morphology of QDs.

**Authors Response:** Thank you for pointing this out. Spherical morphology was observed for smaller QDs (6.1 nm and 9.8 nm) while the larger QDs had cubic morphology. This has been highlighted in the manuscript in lines 177 to 179.

**Comment 7:** R3#3: Even if it is a non-biomedical application, the authors need to justify the toxicity or reusability of the coating/coated material.

**Authors Response:** Given that our quantum dots contain lead, we have opted not to pursue additional toxicity investigations for this study. We acknowledge the guidance from the United States Centers for Disease Control and Prevention (CDC), which indicates that no level of lead exposure is entirely safe, especially for children, with a recommended Blood Lead Reference Value (BLRV) of 3.5 micrograms of lead per deciliter of blood (μg/dL). In light of these considerations, we have assumed potential toxicity of the active material and recommend that the proposed layers be used exclusively for non-biomedical applications.

**Comment 8:** Modify the FTIR figures to transmittance.

**Authors Response:** The absorbance spectra have been changed into transmittance for Figures 3 and 7.

**Reviewer 2**

**No comments.**

**Reviewer 3**

**Comment 1:** I appreciate the authors for addressing the comments. However, a single SEM image and EDX result are insufficient to conclusively verify the formation, morphology, and quality of PbTe QDs. Additional SEM images and X-ray diffraction (XRD) must be performed to provide a comprehensive characterization of the PbTe QDs.

**Authors Response:** Thank you for your comment. A detailed report on the fabrication and characterization of the PbTe layers as used in our experiments was given in Lyssenko et al., 2025 ([doi.org/10.1371/journal.pone.0317677](https://doi.org/10.1371/journal.pone.0317677.g001)), including XRD results showing patterns matching those previously reported in literature for PbTe. This publication has been referenced in our characterization section to provide readers with more complete information about the fabricated layers.

Abuh Samuel Onuh,

Department of Physics,

Ariel University, Israel.
