# Supplementary material for: Antibacterial property of lead telluride quantum dot layer fabricated on glass substrate
Source: PLoS One. 2025 Oct 16;20(10):e0334629. doi: 10.1371/journal.pone.0334629 (PMC12530537; doi:10.1371/journal.pone.0334629)
Supplement: Attachment — Submitted filename: Response_To_Reviewers_auresp_3.docx [file pone.0334629.s008.docx]

**Response to Reviewers’ Comments on Manuscript PONE-D-24-42235R2**

The Academic Editor

*PLOS ONE.*

Dear Dr. Thakur,

Thank you for giving us the opportunity to submit a revision of the manuscript ‘Antibacterial Property of Lead Telluride Quantum Dot Layer Fabricated on Glass Substrate’ (PONE-D-24-42235R2) for publication in *Plos One.* The concern you raised regarding the reference list was noted and the list has been reformatted to follow the *Plos One* style requirement. Please see below our response to the reviewers’ comments.

Review Comments for the Authors

**Reviewer 1**

**Comment 1:** The authors have included a citation to all the comments by R#1. Although more evidence is needed, the justification might be sufficient for the manuscript to be accepted.

**Author Response:** We thank you for recommending our manuscript for publication. We also believe that additional evidence is needed, especially with respect to the proposed application for PbTe QDs layers. However, we note that our investigation is preliminary and presents results that center primarily on the ability or otherwise of PbTe layers to inhibit bacteria growth.

**Reviewer 2**

**Comment 1:** Thank you for the opportunity to review the *PLOS ONE* manuscript. The authors have revised the manuscript according to the comments and I recommend that the manuscript be accepted for publication.

**Author Response:**

We thank the reviewer for recommending our manuscript for publication.

**Reviewer 3**

(No Comment)

Abuh Samuel Onuh,

Department of Physics,

Ariel University, Israel.
